# MiR 329/449 Suppresses Cell Proliferation, Migration and Synergistically Sensitizes GBM to TMZ by Inhibiting Src/FAK, NF-kB, and Cyclin D1 Activity

**DOI:** 10.3390/ijms26125533

**Published:** 2025-06-10

**Authors:** Megan Mendieta, Mehrdad Bandegi, Ezgi Biltekin, Yasemin M. Akay, Bulent Ozpolat, Metin Akay

**Affiliations:** 1 Biomedical Engineering Department, University of Houston, Houston, TX 77204, USA; mmendieta@uh.edu (M.M.); mbandegi@cougarnet.uh.edu (M.B.); ebiltekin@houstonmethodist.org (E.B.); makay@uh.edu (M.A.); 2 Department of Nanomedicine, Houston Methodist Research Institute, Houston, TX 77030, USA

**Keywords:** GBM, spheroids, miRNA, drug resistance, migration, synergy, sensitization

## Abstract

Glioblastoma Multiforme (GBM) is one of the most common brain tumors and is associated with aggressive tumor characteristics and extremely poor patient survival. The median survival time for GBM patients is around 12–15 months. Temozolomide (TMZ) is a key chemotherapeutic drug used in the treatment of GBM. However, at least 50% of GBM patients do not respond to TMZ, necessitating the identification of novel therapeutic strategies sensitizing patients to TMZ. In this study, we aimed to investigate the effects of two different tumor suppressor microRNAs (miR-329 and miR-449b) on cell proliferation and migration of GBM cells, and their potential for sensitizing GBM cells to TMZ. Our findings show that MiR-329/449b treatments suppressed spheroid formation and migration of GBM (LN229 and U87) cells. When miR treatments were combined with Temozolomide (TMZ), we also observed that they synergistically enhanced the suppressive effects of TMZ and inhibited the activity of clinically significant NF-KB and Src/FAK signaling pathways, making the combination therapy a viable option to treat GBM, with greater impact on patient survival.

## 1. Introduction

Glioblastoma Multiforme (GBM) is a grade IV astrocytoma, which arises from either glial cells, neural stem cells, or glial progenitor cells, and is a highly aggressive brain tumor [1,2]. GBM displays intra-tumoral heterogeneity in gross appearance and is ultimately pleomorphic, in that multiple cell lineages are present in the same tumor with differing characteristics, such as growth rate and invasiveness [3,4]. Over 90% of glioblastomas multiforme cases form from normal astrocytes through multistep oncogenesis (de novo), which involves aberrations in multiple signaling pathways and genetic mutations [5]. There is an urgent need for novel therapeutic treatments for GBM due to its infiltrative and recurrent nature, as well as the low efficacy of the current treatment strategies [6]. Management of GBM includes surgical resection, chemotherapy, such as with Temozolomide (TMZ), and radiotherapy, with an average survival length of 15 months and the five-year survival rate remaining at around 6.8% [7]. Although Temozolomide (TMZ) is a key chemotherapeutic drug used in the treatment of GBM, about 50% of GBM patients do not respond to it. TMZ resistance is an important clinical issue, contributing to high mortality and poor survival of patients. Therefore, there is an urgent need for novel therapeutic treatments for GBM that work as monotherapies or enhance the efficacy TMZ [6]. Previous studies indicated that NF-kB plays a role in TMZ resistance and becomes upregulated in response to repair methylated DNA [8]. Src has also been observed to become upregulated in response to TMZ administration, modulating proliferation, differentiation, motility, and adhesion [9].

**Figure 1:**
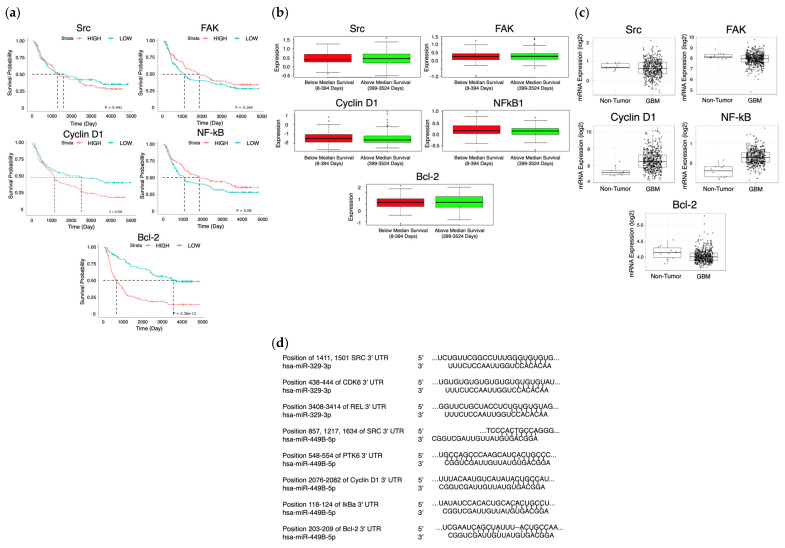
Survival and expression difference in potential proteins and miR targets. The CGGA (n = 325) (**a**) and Glioma-BioDP (n = 264) (**b**) platforms were utilized to determine differential survival probability between high and low expressing patients for Src, FAK, Cyclin D1 (CCND1), NF-kB, and Bcl-2. The GlioVis platform (using TGCA data) (**c**) allowed comparison of expression of Src, FAK, Cyclin D1 (CCND1), NF-kB, and Bcl-2 in normal tissue to expression in GBM. Prediction of 3′ UTR binding sites for miRNA on AXL and eEF2K mRNA (**d**) was made with TargetScan and miRDB.

Dysregulation of NF- kB signaling can promote oncogenesis via the inhibition of antitumor immune responses, and can transcriptionally activate the Bcl-2 family [10]. Specific cell survival pathways in cancers become activated to promote cell survival, such as the FAK/Src pathway, which is closely involved in cell cycle progression and proliferation in that FAK signaling can lead to increased cyclin D1 expression, a key regulator of the G1 to S phase transition [11]. Src and NF-kB have been repeatedly implicated as poor prognostic factors in many cancers, including triple negative breast cancer, and we found that their upregulation has been shown to decrease patient survival in GBM (Figure 1a,b) [12]. This data correlates with increased oncogenic activity of these proteins in GBM patient cells in comparison to healthy brain tissue, where oncogenic proteins are relevant targets for miR treatment (Figure 1c). In oncogenic proteins which do not appear to demonstrate decreased survival or differential expression in GBM versus healthy tissues, it is pertinent to understand and elucidate the impact of phosphorylated protein activity on each.

MicroRNAs (miRs) have been shown to play a role in the pathogenesis of many human cancers, by contributing tumor growth, invasion, metastasis, drug resistance, angiogenesis and progression in solid tumors and GBM [13,14]. MicroRNAs are small, non-coding regulatory RNA genes, which target messenger RNA (mRNA) degradation and suppression of protein translation [15,16]. The MiR expression level may serve as a biomarker and target for novel tumor treatment, in that its dysregulation allows tumors to obtain and sustain the malignant hallmarks of cancer [17]. Tumor suppressor miRs are thought to be beneficial as a potential cancer treatment in that they can specifically target and suppress known oncogenes and, in turn, modulate cellular response to standard therapy regimens, restoring tumor cell sensitivity [18].

It is well known that 2D cell culture models cannot fully recapitulate the complex microenvironment in which a tumor resides, whereas 3D models, which are reproducible, can be utilized to fine tune the tumor microenvironment for screening of therapeutics [19]. A 3D poly(ethylene glycol) dimethyl acrylate (PEGDA) platform, which mimics in vivo behaviors of tumor cells to develop potential cancer therapies, was previously developed in our lab [20,21]. The platform was successfully applied to the investigation of pertinent cell-to-cell interactions, including that of astrocytes and endothelial cells, affecting the efficacy of treatment strategies [22,23]. It was also used to validate the findings generated in the cell-to-cell interaction studies by correlating treatment efficacy with a novel biomarker, tumor stiffness, which is only made possible in 3D culture [24]. It is imperative that, when considering drug development pipelines, proper modeling of tumor features is of the utmost priority, which is why our microwells are employed in this study to accurately observe the possible advantages of emerging miR treatments.

In this study, we further interrogated the link between miR restoration and the inhibition of proliferative capabilities, in LN229 and U87 GBM cell lines, and sensitization to TMZ. Our findings suggest that these miR treatments reduce proliferative and migratory capabilities of LN299 and U87, in synergy with the antiproliferative effects of TMZ by downregulating Src/FAK, and Cyclin D1 pathways, subsequently leading to increased apoptosis in both GBM cell lines. The effect of miR treatments with TMZ requires further investigation to better understand its underlying mechanisms in GBM and its potential to be delivered in synergy with existing therapeutics for GBM treatment that could increase the length of survival for patients.

## 2. Results

### 2.1. MiR-329 and miR-449b Expression Impairs GBM Cell Migration

**Figure 2:**
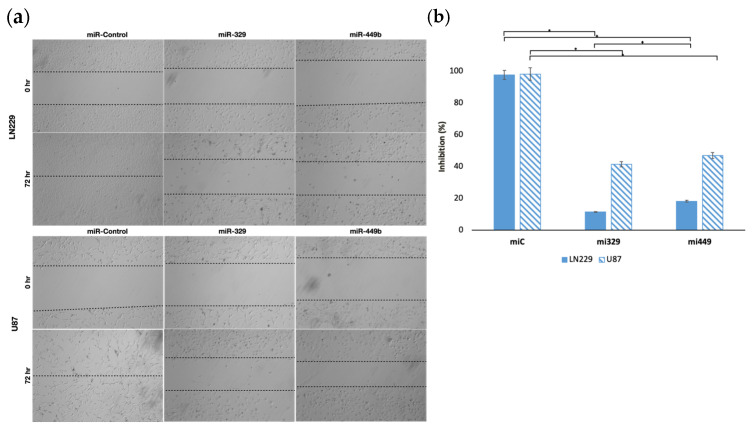
Representative images of miR treated GBM cell lines in a wound-healing assay. The migration capacity of U87 and LN229 cells lines (**a**), transfected with controls or miRNA, was observed after introducing a scratch into each cell monolayer. The area of the wounds were measured at 0 hr and 72 hr and the percentage difference between timepoints (**b**) was calculated. Data represent the mean ± SD of three biological replicates. * = *p* < 0.05.

Based on our findings we decided to first investigate the ability of the selected miRs to target motility regulating proteins with differential survival and expression in GBM patients. We hypothesized that miR-329 and miR-449 would be able to inhibit the motility, and therefore migration capacity, of the LN229 and U87 cell lines in a wound healing assay, due to the rescue of miR-329 and miR-449b expression (Figure 2a). To define this effect, we assessed wound healing in both cell lines when transiently transfected with control miRNA, miR-329 or miR-449. MiR-329 and miR-449b expression significantly impaired GBM cell migration compared to the control condition. In LN229 cells, miR-329 transfection resulted in an 88.3% reduction in wound closure, while miR-449b reduced migration by 81.5% relative to the control (Figure 2b) (*p* < 0.05). Similarly, in U87 cells, miR-329 and miR-449b treatments led to a 57.9% and 52.2% decrease in migration capacity, respectively, compared to the control (*p* < 0.05). Notably, LN229 cells exhibited a more pronounced reduction in migration upon miRNA treatment than U87 cells, suggesting potential cell line-specific differences.

### 2.2. Synergistic Concentrations of TMZ and miR Treatments Optimized for 3D Applications in GBM

**Figure 3:**
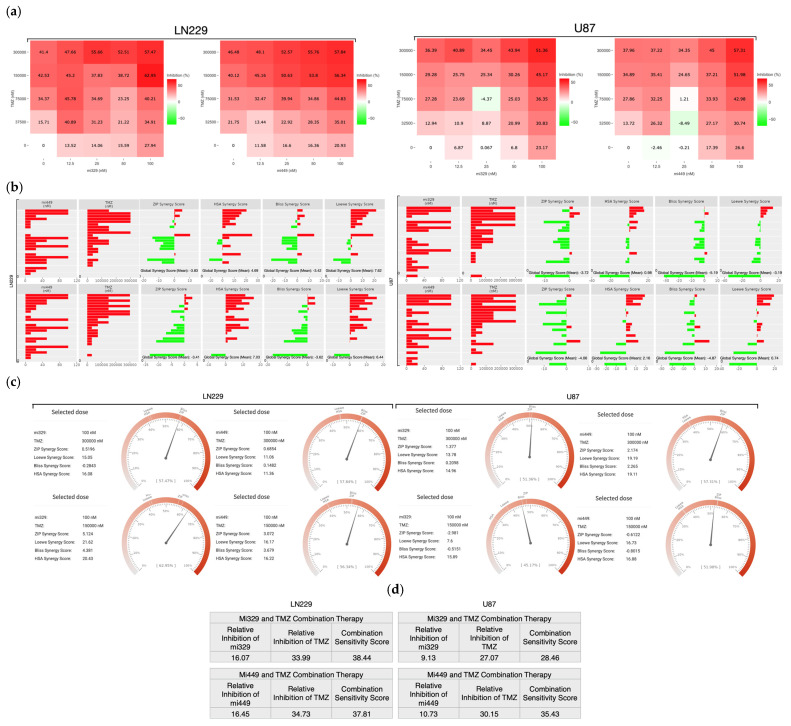
Optimization of TMZ and miR combinations for LN229 and U87 spheroid treatment. TMZ was diluted at a 1:2 ratio from 300 µM, and miR-329 and mi-449 were diluted at a 1:2 ratio from 100 nM. U87 and LN229 cells were cultured in monolayer until confluent and transfected with miR-329 or miR-449b at a specific dilution. After 3 days they were collected and seeded in low adhesion u-bottom plates to form spheroids for 3 days. Spheroids were then treated with TMZ at a specific dilution for 48 h and then a luminescent reagent was applied to determine inhibition percentage (**a**) for each drug combination by normalizing to the control and subtracting the determined cell viability from 100%. ZIP, BLISS, HSA, and LOEWE synergy models (**b**) were applied to the matrix of inhibition percentages for each cell line and TMZ-miR drug combination. Individual combinations with the highest synergy scores (**c**) were identified and overall drug pair sensitivity (**d**) determined. Data represent the mean ± SD of three biological replicates.

To determine the specific drug concentrations at which to treat 3D cultured GBM cell lines, we designed a synergy assay combining the effects of TMZ and miR treatments. The synergy assay was designed with previously validated concentrations of TMZ (300 µM) and miRNA (100 nM) in mind for the maximum doses [25]. From these maximum doses, dilutions were created, each decreasing at a 1:2 ratio, with the inclusion of untreated controls. From each cell line, 3D steroids were formed from miR-329 or miR-449b transfected cells (or not transfected cells) in low-attachment plates and then treated (or not) with standard chemotherapeutic TMZ. These spheroids were probed with a CellTiter-Glo 3D reagent, which causes a luciferase reaction, generating a luminescent signal proportional to the amount of ATP present in the cells. This resulted in a matrix of inhibition percentage (Figure 3a) across all combinations, performed in triplicate (*p* < 0.05). SynergyFinder+ was utilized to generate synergy values across all combinations and means of each two-drug combination, in both LN229 and U87 cell lines [26]. The ZIP model generates the expected effect of two drugs by assuming they do not potentiate each other [27]. The Highest Single Agent (HSA) model investigates whether the expected effect of the combination of two drugs is equal to the higher effect of the individual drugs [28]. The Bliss model assumes the two drugs exert their effects independently, and the expected effect of the combination can be calculated based on the probability of such independent events [29]. The Loewe model considers the dose–response curves of individual drugs and defines the expected combination effect is if the each of the drugs were combined with themselves [30]. Having all four models available (Figure 3b) to evaluate the synergy of the drugs provides a larger and more nuanced picture of the overall effect of the combined drugs, where scores from 0 to 10 indicate additive effects, negative scores indicate antagonism, and scores greater than 10 indicate synergism [31]. The global mean synergy of LN229 spheroids treated by miR-329 was 4.69 and 7.62 for HSA and Loewe models, respectively, but −3.83 and −3.42 for ZIP and Bliss, respectively (*p* < 0.5). For LN229 spheroids treated by miR-449b, the global mean synergy was 7.03 and 6.44 for HSA and Loewe models, respectively, but −3.41 and −3.62 for ZIP and Bliss, respectively (*p* < 0.05). For U87 spheroids treated by miR-329, the global mean synergy was 0.98 and −3.19 for HSA and Loewe models, respectively, but −3.72 and −5.19 for ZIP and Bliss, respectively (*p* < 0.05). Lastly, for U87 spheroids treated by miR-449b, the global mean synergy was 2.16 and 0.76 for HSA and Loewe models, respectively, but −4.06 and −4.87 for ZIP and Bliss, respectively (*p* < 0.5). This is indicative of the drugs having some synergy, but either similar effects or mechanistic interference at non-ideal doses, which is why we then further investigated for drug synergy at more precise concentrations.

Largely for both cell lines, the combination of 100 nM of miR-329 or miR-449b and 300 µM TMZ (Figure 3c) was the most synergistic, or was at least highly additive in effect. The highest synergy scores for 100 nM of miR-329 and 300 µM TMZ or 100nM of miR-449b and 300 µM TMZ in the LN229 cell line were, respectively, 16.08 (HSA) and 11.36 (HSA) (*p* < 0.05). The highest synergy scores for 100 nM of miR-329 and 300 µM TMZ or miR-449b and 300 µM TMZ in the U87 cell line were, respectively, 14.96 (HSA) and 19.19 (Loewe) (*p* < 0.05). The ZIP and Bliss models for this combination of concentrations in both cell lines followed the previous global trend in demonstrating decreased synergy values but, in this more specific case, were at least reflective of additive effects (*p* < 0.05).

Relative inhibition (RI) is the proportion of the area under the dose–response curve, to the maximum area that one of the drugs can reach at the same dose range. The Combination Sensitivity Score (CSS) is a metric used to determine the sensitivity of a drug combination by determining the overall inhibition of a drug combination based on the area under the dose–response curves at IC-50 doses [32]. If the RI of each constituent drug is individually lower than the CSS, the drug is assumed to be synergistic. When evaluating the CSS in each drug in the combination for both cell lines (Figure 3d), we found all RI values to be lower than their subsequent CSS values (*p* < 0.05). For mir-329 and TMZ treated LN229 spheroids, the respective drug RIs were 16.07 and 33.99, with a CSS value of 38.44. When considering mir-449b- and TMZ-treated LN229 spheroids, the respective drug RIs were 16.45 and 34.73, with a CSS value of 37.81. For mir-329- and TMZ-treated U87 spheroids, the respective drug RIs were 9.13 and 27.07, with a CSS value of 28.46. When considering mir-449b- and TMZ-treated U87 spheroids, the respective drug RIs were 10.73 and 30.15, with a CSS value of 35.43. There were several highly synergistic drug concentrations but, for consistency, 100 nM of miR and 300 µM of TMZ were selected for the remainder of the experiments to determine differential cell viability and expression.

### 2.3. Synergistic Application of TMZ and miR Treatments to PEGDA Microwell GBM Spheroids’ Reduced Viability of GBM Cells

**Figure 4:**
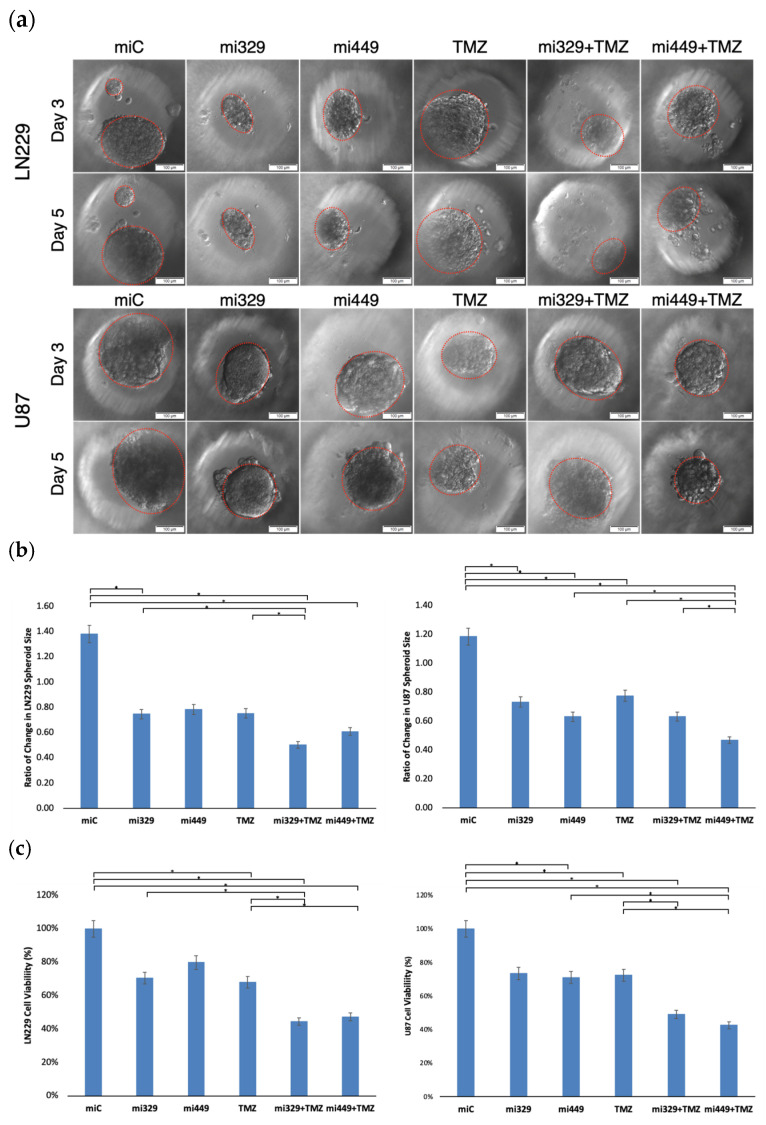
Representative images of miR- and/or TMZ-treated GBM cell lines in 3D PEGDA microwells. U87 and LN229 cells were cultured in monolayer until confluent and transfected with no drug, control miRNA, miR-329 or miR-449b at a synergistic concentration. After 3 days, they were collected and seeded in PEGDA microwells to form an array of spheroids for 3 days (**a**). Spheroids in the microwells were then treated with no drug or TMZ for 48 h and then the average spheroid area ratio (**b**) and cell viability (**c**) were calculated. Data represent the mean ± SD of three biological replicates. * = *p* < 0.05.

To analyze the effects of the determined synergistic concentrations of miR-329 or miR-449b and TMZ, LN229 or U87 cells were transfected at 100 nM and then cultured in 3D PEGDA microwell chips, as seen in Figure 4a. After three days of spheroid formation, TMZ was applied to the microwells (300 µM) and the spheroids were left to incubate for two more days. Spheroids were evaluated for changes in area with ImageJ using images taken at day three of spheroid formation before treatment and at day five (Figure 4b). In the LN229 cell line, the miR-Control treatment generated a 38% increase (1.38 ratio) in spheroid area (*p* < 0.05). Treatment with miR-329 and miR-449b alone decreased spheroid area to 75% and 78% of initial size, respectively (*p* < 0.05). TMZ alone induced a reduction to 75% of initial spheroid area (*p* < 0.05). Combination therapies miR-329 with TMZ, and miR-449b with TMZ, resulted in a reduction to 50% and 61% of initial spheroid size, respectively, with both treatments being significantly reduced versus the control (*p* < 0.05). In the U87 cell line, the miR-Control treatment resulted in a 19% increase in spheroid size (*p* < 0.05). Treatment with miR-329 retained 73% of initial size and miR-449b retained 63% of initial size (*p* < 0.05). TMZ alone demonstrated the least inhibition of spheroid growth in that TMZ spheroids remained at 78% of initial size. Combination treatments miR-329 with TMZ at 63% of initial area and miR-449b with TMZ at 47% of initial area, resulted in the most significant reduction in spheroid size (*p* < 0.05). It can also be observed that spheroids treated with miRs by day 3 after seeding, are malformed. Visually, the spheroids demonstrate characteristics that imply a decreased ability to create tight cell-to-cell interactions, resulting in a less defined boundary.

Spheroids were also evaluated for cell viability using the trypan blue staining assay (Figure 4c). Cell viability in the LN229 cell line was normalized to miR-Control treatment. Treatment with miR-329 and miR-449b significantly decreased cell viability (*p* < 0.05), with miR-329 lowering viability to about 70% and miR-449b to about 80%, while a statistically comparable reduction was recorded for TMZ alone at 68%. Among all treatments, the combination therapies miR-329 with TMZ and miR-449b with TMZ led to the largest reduction in cell viability, dropping to 44% and 47% viability, respectively, which was statistically significantly lower than that for the control and TMZ (*p* < 0.05). In the U87 cell line, viability was also normalized to the control treatment. Treatment with miR-329 and miR-449b alone caused significant reductions in cell viability to 73% and 71% (*p* < 0.05). TMZ alone moderately affected cell viability, resulting in a decrease to 72%. The combination treatments significantly reduced U87 cell viability (*p* < 0.05), where miR-329 with TMZ decreased viability to 49%, but the greatest effect was observed in miR-449b with TMZ at 43% (*p* < 0.05). Spheroid size and cell viability studies generally demonstrated correlating results, which further confirms the effect of the treatments.

### 2.4. Src/Fak Pathway Downregulated in GBM Spheroids by Synergistic Concentrations of TMZ and miR Treatments

**Figure 5:**
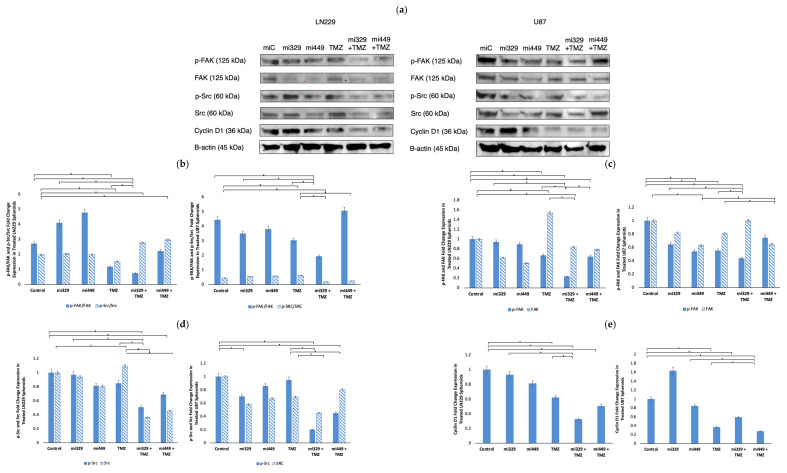
Effect of miR and TMZ synergy on Src/Fak signaling pathways in GBM spheroids. Western blot analysis was conducted on U87 and LN229 spheroids, which were first cultured in monolayer until confluent, and transfected with no drug, control miRNA, miR-329 or miR-449b at a synergistic concentration. After 3 days, they were collected and seeded in PEGDA microwells to form an array of spheroids for 3 days. Spheroids in the microwells were then treated with no drug or TMZ for 48 h and then the cells were collected and their protein processed for immunoblotting (**a**). Ratios of comparative expression for p-FAK/FAK and p-Src/Src (**b**) were calculated by normalizing to B-actin expression and then, individually, p-FAK and FAK (**c**), as well as p-SRC, and Src (**d**) fold change expression was quantified and normalized to B-actin and the control sample expression. Finally, Cyclin D1 (**e**) fold change expression was quantified and normalized to B-actin and the control sample expression. Data represent the mean ± SD of three biological replicates. * = *p* < 0.05.

To further confirm whether the Src/FAK signaling pathway was downregulated by the combination of miR-329 or miR-449b and TMZ, the expression of Cyclin D1, p-FAK, FAK, p-Src, and Src, which regulate cell cycle progression and adhesion, were investigated. Cyclin D1, which is a key regulator of the G1-to-S phase transition [33], was significantly downregulated in co-treated groups. In LN229 spheroids, Cyclin D1 expression was reduced to 0.33 in the miR-329 with TMZ treatment group and 0.50 in the miR-449b with TMZ treatment group compared to the control treatment (*p* < 0.05) (Figure 5e). In U87 spheroids, Cyclin D1 levels were 0.59 in miR-329 with TMZ treatment groups and 0.28 in miR-449b with TMZ treatment groups (*p* < 0.05).

p-FAK and p-Src, two signaling proteins involved in GBM migration and invasion, were also investigated following co-treatment. In LN229 spheroids, p-FAK expression (normalized to the control) was reduced to 0.23 in miR-329 with TMZ treatment groups and 0.64 in miR-449b with TMZ treatment groups while p-Src levels (normalized to the control) dropped to 0.51 and 0.69, respectively (*p* < 0.05) (Figure 5d). In U87 spheroids, p-FAK expression significantly decreased in the miR-329 with TMZ group to less than 0.50 and in the miR-449b with TMZ group to 0.74 compared to control treatment (*p* < 0.05) (Figure 5c). p-Src expression in U87 was also suppressed in the combination treatment groups, with miR-329 with TMZ at 0.20 and miR-449b with TMZ at 0.45 (*p* < 0.05). FAK and Src expression in both cell lines also decreased in co-treatment groups, with some of the largest values of down regulation coming from solo miR drug treatment, affecting phosphorylated protein to total protein ratios (Figure 5c,d). MiR-329 with TMZ treatment groups had the largest effect in LN229 for p-FAK/FAK and p-Src/Src at 0.74 and 2.78 respectively. In U87 for p-FAK/FAK and p-Src/Src, ratios from the MiR-329 with TMZ treatment groups decreased 1.93 and 0.20, respectively (*p* < 0.05).

### 2.5. NF-kB Activity Downregulated in GBM Spheroids by Synergistic Concentrations of TMZ and miR Treatments

**Figure 6:**
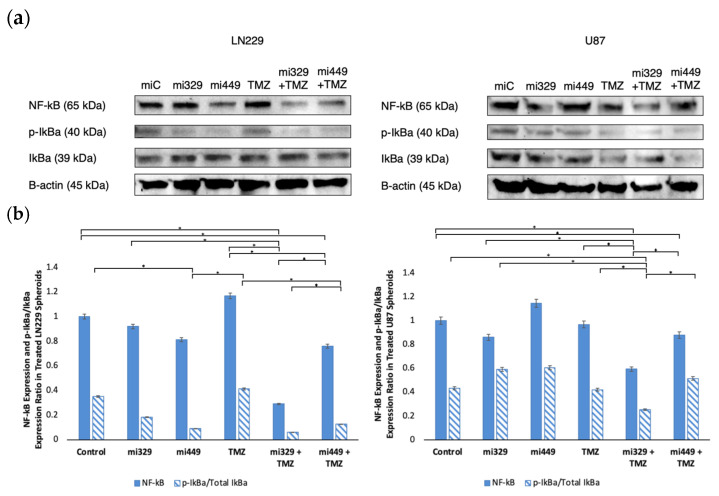
Effect of miR and TMZ synergy on the NF-kB signaling pathway in GBM spheroids. Western blot analysis was conducted on U87 and LN229 spheroids, which were first cultured in monolayer until confluent and transfected with no drug, control miRNA, miR-329 or miR-449b at a synergistic concentration. After 3 days, they were collected and seeded in PEGDA microwells to form an array of spheroids for 3 days. Spheroids in the microwells were then treated with no drug or TMZ for 48 h and then the cells were collected and their protein processed for immunoblotting (**a**). NF-kB, p-IkBα, and IkBα (**b**) fold change expression was quantified and normalized to B-actin and NF-kB was also normalized to the control. Data represent the mean ± SD of three biological replicates. * = *p* < 0.05.

To probe NF-κB pathways after drug treatment, we investigated the expression of NF-κB in both treated and untreated U87 and LN229 GBM spheroids. Please note that loading controls were used for each experiment, but only the representative loading control was displayed (Figure 6a). For all treatments and both cell lines, NF-κB expression was normalized to the miR-Control treatment expression. In LN229 spheroids (Figure 6b), treatment with miR-329 and miR-449b alone reduced NF-κB expression to 92% and 81%, respectively, when normalized to the control levels (*p* < 0.05). TMZ treatment alone led to a 17% increase in NF-κB expression, while the miR-329 with TMZ and miR-449b with TMZ co-treatments resulted in significant reductions to 29% and 76%, respectively, when normalized to the control levels (*p* < 0.05). In U87 spheroids, miR-329 and miR-449b alone led to NF-κB expressions of 86% and 115%, respectively, when normalized to the control levels. TMZ treatment alone resulted in 97% NF-κB expression, while miR-329 with TMZ and miR-449b with TMZ co-treatments resulted in reductions to 59% and 88%, respectively, normalized to the control (*p* < 0.05).

We also analyzed the p-IkBα/Total IkBα expression ratio as a measure of NF-κB signaling activation (Figure 6b). For all treatments and both cell lines, p-IkBα and total IkBα expression was normalized to the miR-Control treatment expression. In LN229 spheroids, treatment with control miR, miR-329, and miR-449b alone led to ratios of 0.35, 0.18, and 0.09, respectively. TMZ alone resulted in a ratio of 0.41, while miR-329 with TMZ and miR-449b with TMZ co-treatments further decreased the ratio to 0.06 and 0.13, respectively (*p* < 0.05). In U87 spheroids, treatment with control miR, miR-329 and miR-449b alone led to ratios of 0.43, 0.59, and 0.61, respectively. TMZ treatment alone resulted in a ratio of 0.42, while miR-329 with TMZ and miR-449b with TMZ co-treatments led to reductions to 0.25 and 0.51, respectively (*p* < 0.05). These findings suggest that the synergistic combinations of miR-329 or mirR-449b and TMZ offer a potential therapeutic strategy to modulate NF-κB activity in glioblastoma and, therefore, therapeutic resistance.

### 2.6. Apoptosis Signaling Promoted in GBM Spheroids by Synergistic Concentrations of TMZ and miR Treatments

**Figure 7:**
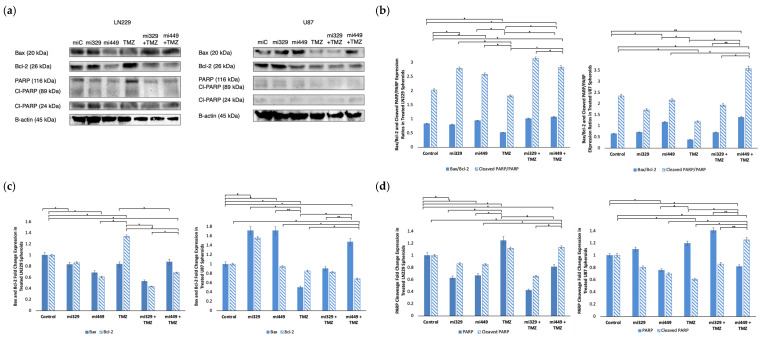
Effect of miR and TMZ synergy on the intrinsic and extrinsic apoptotic signaling pathways in GBM spheroids. Western blot analysis was conducted on U87 and LN229 spheroids, which were first cultured in monolayer until confluent and transfected with no drug, control miRNA, miR-329 or miR-449b at a synergistic concentration. After 3 days, they were collected and seeded in PEGDA microwells to form an array of spheroids for 3 days. Spheroids in the microwells were then treated with no drug or TMZ for 48 h and then the cells were collected and their protein processed for immunoblotting (**a**). Expression ratios for Bax/Bcl-2 and Cleaved PARP/PARP (**b**) were calculated by normalizing to B-actin expression and then, individually, Bax and Bcl-2 (**c**) as well as PARP and Cleaved PARP (**d**) fold change expression were quantified and normalized to B-actin and the control sample expression. Data represent the mean ± SD of three biological replicates. * = *p* < 0.05 and ** = *p* < 0.01.

To probe whether apoptosis signaling was induced by co-treatment, we assessed the expression of intrinsic and extrinsic apoptotic markers, including Bax, Bcl-2, PARP, and cleaved PARP, in U87 and LN229 GBM spheroids. The Bax/Bcl-2 ratio serves as a crucial indicator of intrinsic apoptotic susceptibility, as well as of the balance of PARP and its cleavage. Please note that loading controls were used for each western blot experiment, but only the representative loading control was displayed (Figure 7a). In LN229 spheroids, the control group had a baseline Bax/Bcl-2 ratio of 0.83, while treatment with miR-329 and miR-449b alone resulted in ratios of 0.80 and 0.94, respectively (Figure 7b). TMZ alone led to a decrease in the ratio to 0.52, whereas miR-329 with TMZ and miR-449b with TMZ co-treatments increased the ratio to 1.01 and 1.07, respectively (*p* < 0.05). In U87 spheroids, the Bax/Bcl-2 ratio was 0.65 in the control group, 0.71 with miR-329 and 1.17 with miR-449b (*p* < 0.05). TMZ alone reduced the ratio to 0.38, while the combination treatments of miR-329 with TMZ and miR-449b with TMZ resulted in ratios of 0.71 and 1.39, respectively. Upon analyzing Bcl-2 expression alone in the miR-329 with TMZ and miR-449b with TMZ co-treatments, in both spheroid cell line models were down regulated, to 0.44 and 0.69 respectively, compared to the control and single-drug treatments (*p* < 0.05) (Figure 7c). Bax expression was relatively unchanged in LN229 across treatments. However, in U87, Bax expression was increased in the miR-329 to 1.72 and in miR-449b to 1.72, with a moderate increase observed in miR-449b with TMZ to 1.47 (*p* < 0.05).

To further assess apoptosis, we analyzed the cleaved PARP/PARP expression ratio as an indicator of caspase-dependent cell death (extrinsic). Western blot analysis revealed that the cleaved PARP/PARP expression ratio was highest in co-treatments (Figure 7b), miR-329 with TMZ at 3.15 and miR-449b with TMZ at 2.83 in LN229, compared to the control treatment value of 2.03 (*p* < 0.01). In U87, the cleaved PARP/PARP expression ratios were similarly increased in the miR-449b with TMZ group to 3.59, in comparison to the control treatment value of 2.35 (*p* < 0.01). These findings were consistent with total PARP levels (Figure 7c), which were significantly decreased in LN229 and U87 spheroids following co-treatment compared to control and TMZ treatments (*p* < 0.05).

## 3. Discussion

Glioblastoma Multiforme remains one of the most aggressive and treatment-resistant brain malignancies, with current standard-of-care therapies, including TMZ, offering only limited improvements in patient survival. Due to the high failure rates and extended development timelines associated with novel drug discovery, repurposing existing therapeutic agents and exploring innovative combinatorial approaches are of significant interest. MicroRNAs have emerged as promising candidates for enhancing sensitivity to TMZ, given their role in regulating key oncogenic pathways. MiR-329 and miR-449b have been shown in previous studies to be under-expressed versus healthy tumors, leading to shorter survival in patients, and are thought to be complementary to the mRNA of oncogenic proteins, such as AXL, eEF2K, Src, Fak, Cyclin D1, NF-kB, and Bcl-2 [34]. MiR-449b acts as a tumor suppressor, inhibiting cell proliferation and slowing down oncogenesis by inactivating the Wnt/β-catenin signaling pathway [35]. Upon analyzing the Cancer Genome Atlas (CGA), miRDB, and TargetScan, it was determined that miR-329b and 449b are associated, and these miR sequences have a significant probability to target more proteins directly within the cascade, such as FAK, Src, Cyclin D1, NF-kB, and BCL-2 (Figure 1d) [36,37]. If GBM patients with poorer survival outcomes also tend to have lower miR-329 and miR-449b tumor expression, increased expression or delivery of these miRs to GBM cells can lead to tumor cell sensitization and decreased motility and proliferation [38].

In our initial wound healing assay, the migration capacity of monocultured U87 and LN229 cell lines was interrogated. It was confirmed that miR-329 and miR-449b were able to inhibit the motility of these cell lines, where LN229 was most affected by each treatment, possibly demonstrating increased resistance in the U87 cell line. Previous studies have indicated that the LN229 and U87 cell lines exhibit differentially reduced expression of miR-329 and miR449b from normal brain tissue (healthy astrocytes) but also have differential expression when compared to each other, with LN229 displaying slightly lower expression of both miRs [39,40,41].

We then investigated the effects of miR-329 with TMZ and miR-449b with TMZ in 3D cultured GBM spheroids from the U87 and LN229 cell lines, employing four different synergy models to assess their potential therapeutic value. Our results demonstrated synergy, which was model-dependent, with the Loewe and HSA models indicating strong combinatorial effects, while ZIP and Bliss models suggested possible mechanistic interference or non-ideal dose ratios. The largest synergistic scores were observed at 100 nM miRNA and 300 µM TMZ, from HSA and Loewe models, with LN229 showing more sensitivity to miR-329 and TMZ and U87 showing more sensitivity to miR-449b and TMZ. The Combination Sensitivity Score supported these findings, as the inhibition values for solo drugs were lower than their respective scores when combined. This highlights the importance of dose optimization to achieve maximum therapeutic benefit and avoid non-ideal interactions. Further study into these mechanistic interactions, such as examining protein expression as we do in this research, is important for refining combinatorial dosing strategies and improving clinical translatability.

Following the determination of synergistic concentrations for the selected miRs and TMZ, we applied our findings to the PEGDA microwell 3D spheroid assays. MiR-329 and miR-449b significantly enhanced TMZ efficacy in 3D GBM spheroids. In LN229 spheroids, miR-329 with TMZ treatment led to a 50% reduction in spheroid size and a 44% reduction in viability, while miR-449b with TMZ in U87 spheroids reduced spheroid size by 47% and viability by 43% (*p* < 0.05). These findings again emphasize cell line-specific responses and reinforce the importance of patient-derived models for personalized therapeutic development. The cell lines exhibit differential therapeutic responses due to their difference in underlying baseline gene expression; they are derived from different donor patients with different tumoral cell genetic populations. In our lab, we have previously noted that U87 spheroids in the presence of NF-kB inhibitors have been more resistant to treatment than LN229 spheroids and may more closely mimic effects seen in patient-derived spheroids [42]. Given that 3D spheroids better mimic in vivo tumor activity and drug penetration compared to traditional 2D cultures, these results provide a more physiologically relevant assessment of treatment efficacy, better recapitulating protein expression and cell-to-cell interactions [43].

Western blot analysis elucidated the molecular mechanisms underlying the observed synergy and confirmed the correlation between miR-329, miR-449b, and their mRNA targets. The combination of miR-329 with TMZ or miR-449b with TMZ significantly downregulated expression of p-FAK/FAK, p-Src/Src, Cyclin D1, NF-kB, and Bcl-2. Previous studies using LN229 and U87 cell lines in monolayer have showed that each cell line exhibits differential expression of Src and FAK at baseline, but expression of Src was significantly reduced both in the U87 and LN229 cells and subsequently resulted in decreased phosphorylation of downstream protein, FAK [44]. Other studies have found elevated Src activity in GBM compared with normal brain samples [45]. In our study, the concurrent downregulation of p-FAK and p-Src, which are implicated in GBM migration and invasion, indicates that miRNA–TMZ co-treatment may also hinder tumor cell motility. Total FAK and Src were also downregulated, suggesting that the miRs may have directly inhibited their mRNA from translation in the 3′ untranslated region (3′UTR), in agreement with our earlier miR target findings. Src aberrant activity is also known to be responsible for the stabilization and activation of transcription factors, including NF-κB [46]. Our Src/FAK findings may also explain the decrease in ability for spheroids to form in a tightly bound manner. It is well known that hypoxia is a driver of metastasis and invasion; it would be important to continue investigating the effects of these miRs on this pathway [47,48]. The reduction observed in Cyclin D1 expression suggests impaired cell cycle progression, potentially increasing GBM susceptibility to TMZ-induced cytotoxicity.

Our study also demonstrated that the miRNA–TMZ combination modulated NF-κB activity, a pathway known to promote survival and resistance in GBM. The miR-329 with TMZ co-treatment led to a nearly 70% reduction in NF-κB expression in LN229 spheroids and a 40% reduction in U87 spheroids. Our results suggest that co-treatment with TMZ allowed the spheroids to be more sensitized to NF-κB downregulation than in solo miR-329 treatment, which correlates with our earlier findings, where the miR-329 sequence directly targets the mRNA of NF-κB. The suppression of NF-κB activation was further confirmed by a decreased p-IkBα/Total IkBα expression ratio, where reduction in total IkBα was most pronounced in LN229 and U87 spheroids from miR-329 and TMZ co-treatment groups. IkBα was preliminarily identified as a potential additional target of miR-449b, and these results suggest that co-treatment with TMZ allowed spheroids to be more sensitive to a decrease in IκBα expression, particularly in U87 spheroids. This is similar to the effects observed in our lab using NF-kB specific inhibitor drugs, such as Bay-11, that demonstrate increased resistance in the U87 cell line [42]. It is also well known that NF-κB has direct connections to the regulation of Bcl-2 and Cyclin D1 expression to desensitize tumor cells, including in GBM [49]. Drug resistance can still be lowered when NF-kB expression is suppressed, but through another mechanism than p-IkBa/IkBa, such as through NF-kB subunit suppression. Given that NF-κB activation in GBM especially can promote survival signaling and therapeutic resistance, its inhibition could serve as an effective strategy for improving GBM treatment sensitivity.

Analysis of apoptosis markers revealed that miR-329 with TMZ and miR-449b with TMZ treatments enhanced pro-apoptotic (intrinsic and extrinsic) signaling in GBM spheroids. Co-treatment significantly increased the Bax/Bcl-2 ratio and reduced Bcl-2 expression, suggesting an increase in the likelihood of apoptosis. Since Bcl-2 inhibits mitochondrial outer membrane permeabilization and contributes to TMZ resistance, its downregulation indicates that miRNA co-treatment sensitizes GBM cells to apoptosis [50]. This is in agreement with our findings, which suggest that Bcl-2 is the direct target of miR-449b, resulting in the blocking of Bcl-2 mRNA translation and a decrease in Bcl-2 cytosolic availability. The observed increase in cleaved PARP/PARP expression further supports enhanced caspase-3 activity, confirming a commitment toward apoptosis rather than DNA repair and survival [51]. Given that PARP plays a key role in DNA damage repair, its degradation implies that miRNA–TMZ co-treatment intensifies irreparable genomic damage, ultimately promoting apoptosis over the upregulation of survival mechanisms. Apoptosis is mediated by both intrinsic (mitochondrial) and extrinsic (death receptor) pathways, with both converging on caspase activation. While our findings predominantly suggest intrinsic pathway activation, the increase in cleaved PARP also hints at potential involvement of caspase-3-mediated extrinsic apoptosis. As resistance to apoptosis is a hallmark of GBM, the ability of miRNA co-treatment to enhance TMZ-induced cell death may provide a novel strategy to circumvent therapeutic resistance. Future studies should assess whether miRNA co-treatment influences death receptor signaling or caspase-8 activation to further describe its apoptotic mechanisms.

In conclusion, our work demonstrates that miR-329 and miR-449b enhance the therapeutic efficacy of TMZ by reducing motility and spheroid formation, targeting key survival pathways (FAK/Src, and Cyclin D1), modulating NF-κB signaling, and promoting apoptosis in 3D GBM spheroids. These findings provide a strong rationale for further exploration of miRNA-based strategies to improve GBM treatment outcomes, especially as they may confer reduced cytotoxicity. Moving forward, it will be essential to optimize dosing regimens, clarify molecular mechanisms governing miRNA–TMZ interactions, and validate these findings in increasingly complex 3D models, including cells which also confer desensitizing effects, such as astrocytes, and in preclinical in vivo models to assess their translational potential [22,24,52]. By leveraging targeted miRNA-based therapies, future GBM treatments may achieve enhanced tumor suppression, and reduced therapeutic resistance and systemic cytotoxicity, in order to improve patient survival.

## 4. Materials and Methods

### 4.1. Drugs and Reagents

Temozolomide (TMZ) was purchased from Sigma-Aldrich (St. Louis, MO, USA). MiR Control (inert, non-complementary sequence), MiR-329-3p (AACACACCUGGUUAACCUCUUU) and miR-449b-5p (AGGCAGUGUAUUGUUAGCUGGC) were purchased from Thermo Fisher Scientific (Waltham, MA, USA). HiPerFect Transfection reagent was purchased from Qiagen (Germantown, MD, USA). eEF2K, Cyclin D1, p-FAK, p-Src and b-Actin primary antibodies were purchased from Cell Signaling Technology (Danvers, MA, USA), along with secondary anti-rabbit and anti-mouse HRP-conjugated antibodies. AXL primary antibody and anti-goat HRP-conjugated antibody were purchased from R&D Systems (Minneapolis, MN, USA). CellTiter-Glo^®^ 3D Cell Viability Assay was purchased from Promega (Madison, WI, USA).

### 4.2. Cell Lines and Cell Culture

Primary Human Astrocyte cells from the cerebral cortex were cultured up to passage 6, using poly-l-lysine coated flasks, in specialty Astrocyte Medium containing 2% FBS, 1% Astrocyte Growth Serum, and 1% of 10,000 units/mL of Penicillin and 10,000 μg/mL of, all purchased from Sciencell (Carlsbad, CA, USA). GBM cell lines LN229 and U87 were purchased from the American Tissue Culture Collection (Manassas, VA, USA). LN229 and U87 cells were cultured in a cell culture plate up to passage 10, using Gibco Dulbecco’s modified Eagle’s medium (DMEM) supplemented with 10% FBS, and 1% of 100 U/mL penicillin and 0.1 g/mL streptomycin from Thermo Fisher Scientific (Waltham, MA, USA). All cells were stored in a cell culture incubator at 5% CO_2_, 37 °C.

### 4.3. MiR Transfection

U87 and LN229 cells were first cultured in monolayer in 6-wells plates at a concentration of 0.8 × 10^5^ cells/mL in DMEM. Cells were allowed to incubate (5% CO_2_, 37 °C) overnight and then transfected with miRNA treatments or nonsense sequence controls. Transfection was made possible by combining HiPerFect reagent from Qiagen (Germantown, MD, USA) in FBS negative media with an appropriate concentration of miRNA (miR-329 or miR-449b) in FBS negative media at a 1:1 ratio. The HiPerFect and miRNA solution was gently mixed and allowed to incubate at room temperature for 20 min. 250 μL of the combined solution was added dropwise to each assigned well, after first discarding the initial media. 1550 μL of FBS negative media was added to the sidewall of the wells to reach a final volume of 2 mL. 3 h later, 200 μL of FBS was added to the sidewall of the wells.

### 4.4. Optimization, Synergy Assay, and TMZ Administration

For optimization of spheroid diameter in clear 96-well u-bottom ultra-low adherence Nunclon Sphera wells from Thermo Fisher Scientific (Waltham, MA, USA), U87 and LN229 cells were transfected with miC, miR329, or miR449 at final concentrations of 25 nM, 50 nM, and 100 nM in monolayer. Post-transfection, cells were trypsinized, collected, and seeded at densities of 1500, 3000, and 6000 cells in a final volume of 100 μL per well in 96-well U-bottom plates (non-adherent surface). Spheroid formation was tracked by imaging on day 0, 1, and 3 and spheroid diameters were measured. Optimal cell density was determined based on spheroid size, with 1500 cells per well-producing spheroids averaging ~300 µm in diameter by Day 3.

The synergy assay was conducted by creating an array of combinations of a dilution of miR-329 or miR 449b with a dilution of Temozolomide (TMZ). These 1:2 miR dilutions ranged from 100 nM to 12.5 nM and 1:2 TMZ dilutions ranged from 300 μM to 37.5 μM, including a set of controls. Cells were first transfected with an appropriate amount of miRNA in monolayer and then, after 3 days of spheroid formation, treated with an appropriate amount of TMZ. Spheroid cell viability was interrogated with 30 min of incubation with 100 μL of CellTiter-Glo^®^ 3D Cell Viability Assay to each well, purchased from Promega (Madison, WI, USA). SynergyFinder+ (Helsinki, Finland) was utilized to analyze the data using ZIP, HSA, Bliss, and Loewe synergy models.

### 4.5. Microwell Fabrication and Cell Seeding

Poly(ethylene glycol) diacrylate (PEGDA) microwells were fabricated as previously described [20]. Cover glass slides, size 24 × 24 mm^2^, were treated with 3-(Trimethoxy-silyl) propyl methacrylate 98% (TMSPMA) from Life Technologies (Carlsbad, CA, USA). The slides were first layered with a 20 μL of 40% (*w*/*w*) PEGDA (MW 700), 0.2% (*w*/*v*) photo-initiator (PI) 2-hydroxy-2-methyl propiophenone, and Phosphate Buffered Saline (PBS) solution from Life Technologies (Carlsbad, CA, USA). They were then exposed to Lumen Dynamics the OmniCure^®^ Series 2000 from Lumen Dynamics Group Inc. (Mississauga, ON, Canada) for 36 s from 6 inches away. A total of 250 μL of PEGDA solution was then added onto the slide and cured with UV light for 36 s with a photomask from CADart (Bandon, OR, USA) patterned with 1000 μm diameter dots in a grid, on top. Prepared slides were washed and incubated in 6-well plates containing 2 mL of PBS each, overnight. The PBS was discarded, and cells which had either previously been transfected with 100 nM of miRNA or received a media change were trypsinized, collected, and seeded at a concentration of about 0.2 × 10^6^ cells/mL on each microwell chip in cell culture media droplets of 190 μL. Droplets were allowed to incubate at room temperature for 5 min to allow for the cells to settle into microwells. The remaining 1810 μL of cell culture media was added for a final volume of 2 mL per well. The plates were placed in the cell culture incubator (5% CO_2_, 37 °C) and allowed to aggregate for 3 days before receiving additional TMZ treatment of 300 μM or a completed DMEM media change. Spheroids were monitored on treatment day 0, 1, and 2 using a microscope from Olympus (Tokyo, Japan).

### 4.6. Cell Viability and Spheroid Size Quantification

To quantify the viability of the spheroids after drug administration or control treatment, spheroids were removed from the microwells via PBS wash and trypsinization. The cells were diluted and stained with 0.4% trypan blue solution to count using a hemocytometer for 3 replicates. The viability of the cells in each cell line were normalized to their untreated control group. Spheroid post-treatment images taken on treatment day 0 and 2 using a microscope from Olympus (Tokyo, Japan) were analyzed for area change, and the area of spheroids in each cell line and treatment group was normalized to that of their untreated control group.

### 4.7. Wound Healing Assay

LN229 or U87 cells were seeded at a concentration of 0.5 × 10^5^ cells/mL in 6-well plates to a final volume of 2 mL. Cells were allowed to incubate (5% CO_2_, 37 °C) overnight and then transfected with 100 nM concentration of miRNA or controls. The cells were allowed to incubate for 72 h and then a scratch was applied to the wells with a sterile pipette tip. Images were taken of the “wound” with an Olympus microscope (Tokyo, Japan) at day 0, 1, 2, and 3 and area change was analyzed with ImageJ software Version 1.54p 17(NIH). The inhibition percentage of the treatments in each cell line were normalized to their untreated control group.

### 4.8. Western Blot

Monolayer cultured cells were first washed with cold PBS. Synergistically treated spheroids or untreated monolayer cells were then trypsinized at 37 °C for 5 min until detached, collected at a 1:1 ratio with supplemented DMEM or specialty Astrocyte Medium, and centrifuged at 1400 rpm for 5 min. Once the supernatant was removed, cold PBS was applied to wash and the cells were centrifuged again for 3000 rpm for 5 min. Supernatant was removed and an appropriate amount of cold RIPA Lysis buffer supplemented by protease/phosphatase inhibitor cocktail from Sigma Aldrich (St. Louis, MO, USA) was directly applied to the cell pellet. The mixture was vortexed and then placed on ice for 5 min. Once the incubation was complete, the mixture was centrifuged at 1400 rpm for 15 min and only the supernatant was reserved. A Bradford assay was conducted to determine the protein concentration for each of the samples using Bradford Quick-Start Dye Reagent from Bio-Rad (Hercules, CA, USA) and Bovine Serum Albumin (BSA) from Fisher Scientific (Hampton, NH, USA) to create standards. A total of 40 µg of cell lysate from each sample was loaded into 12% Mini-PROTEAN^®^ TGX™ Precast Protein Gels from Bio-Rad (Hercules, CA, USA) for gel electrophoresis and transferred onto methanol-activated PVDF membranes from Thermo Fisher Scientific (Waltham, MA, USA). Membranes were equilibrated and then blocked with 5% milk in Tris Buffered Saline with Tween20 (TBS-T) for 30 min at room temperature. Incubation with primary antibody (1:500–1:1000) was conducted overnight at 4 °C in either 5% milk or 5% BSA in TBS-T, washed 5 times with TBS-T for 5 min each, and then incubated for 1 h at room temperature with secondary antibody (1:1000–2000) in either 5% milk in TBS-T. After 5 washes for 5 min each in TBS-T, the blot was imaged using a 1:1 Ratio of SignalFire™ Plus ECL Reagents. Data were normalized to B-actin. Membranes were then stripped using Restore™ PLUS from Thermo Fisher Scientific (Waltham, MA, USA) for 5 min at room temperature and reprobed for additional proteins.

### 4.9. Statistical Analysis

Unless otherwise stated, all reported results were taken from three independent experiments, performed in triplicate. Statistical comparisons between groups were performed using the unpaired two-tailed Student’s *t*-test, unless specified differently, where a *p* value < 0.05 indicated a statistically significant difference. The data were presented as the mean ± standard deviation.

## Data Availability

The raw data supporting the conclusions of this article will be made available by the authors on request.

## Abbreviations

The following abbreviations are used in this manuscript:

TMZ	Temozolomide
GBM	Glioblastoma
CGGA	Chinese Glioma Genome Atlas
miR	Micro RNA
mRNA	Messenger RNA
PEGDA	Poly(ethylene glycol) dimethyl acrylate
ZIP	Zero interaction potency
HSA	Highest single agent
TMSPMA	3-(Trimethoxy-silyl) propyl methacrylate
PBS	Phosphate buffered solution
BSA	Bovine serum albumin
TBS-T	Tris-buffered saline with polysorbate (tween) 20
